# A combined endovascular and open repair of innominate artery bifurcation pseudoaneurysm: a case report

**DOI:** 10.1186/s12872-024-04043-2

**Published:** 2024-07-17

**Authors:** Youyao Xu, Tao Zhu, Guobin Chen, Senyan Wu, Xiaoyang Li

**Affiliations:** Department of Cardiovascular Surgery, The Quzhou Affiliated Hospital of Wenzhou Medical University, Quzhou People’s Hospital, No. 100 Minjiang Avenue, Quzhou City, 324000 Zhejiang Province PR China

**Keywords:** Innominate artery aneurysm, Hybrid technique, Vertebral artery, Occluder-covered stent-graft, Endovascular repair

## Abstract

**Background:**

Innominate artery aneurysms (IAAs) are rare and may result in rupture, distal arterial embolization, or local compression without timely treatment. Rupture is the most dangerous of these complications. This article reports a case of innominate artery bifurcation pseudoaneurysm.

**Case presentation:**

The patient was a 45-year-old man who was admitted to the emergency department due to chest discomfort. The computed tomographic angiography (CTA) imaging indicated the presence of a 3.6*2.4 cm saccular aneurysm in the bifurcation of the innominate artery, involving both the right proximal subclavian and common carotid arteries. The patient’s vital signs were normal, there was equal blood pressure in the upper arms and no neurological dysfunction was observed. Gadolinium-enhanced magnetic resonance angiography indicated that the circle of Willis was intact. The treatment involved open surgery combined with endovascular therapy. The external carotid artery was first transposed to the right subclavian artery (RSA) and an 8-mm woven Dacron graft was inserted in the middle. The covered stent graft was then placed in the proximal part of the innominate artery to close the entrance of the aneurysm. Lastly, an occluder was implanted at the origin of the RSA. There were no perioperative or postoperative complications. At 1-year follow-up, no aneurysm was observed on CTA and the right vertebral artery was patent.

**Conclusions:**

This study indicated that the combined use of endovascular therapy and open repair surgery is an effective strategy to treat innominate artery bifurcation pseudoaneurysm.

## Introduction

The aortic branch aneurysms account for approximately 3% of arterial aneurysms, of which innominate artery aneurysms (IAAs) are extremely rare [1]. IAAs are predominantly caused by atherosclerotic degeneration along with infection, trauma, tuberculosis, arteritis, syphilis, and connective tissue disorders [1, 2]. IAA-induced rupture is the most dangerous complication with an incidence rate of approximately 11% in untreated IAA patients. Other complications are secondary to thrombosis, distal embolization, or local compression [3, 4]. Currently, open surgical and endovascular repair techniques are being used for treating IAA, where much research on the efficacy of the endovascular repair techniques has been conducted [4]. This case study reports an innominate artery bifurcation pseudoaneurysm patient, who was treated with occluder combined with a covered stent graft and artery bypass.

### Case presentation

#### Patient information

A 45-year-old Chinese man was admitted to the emergency department with chest distress. The patient had a surgical history of partial pancreatectomy due to a car accident 10 years ago. He was a smoker and had no cardiovascular risk factors.

### Clinical findings

At the time of admission, the patient’s right carotid and branchial arteries indicated normal pulses, his body temperature was 36.2 °C, the heart rate was 92 beats/min, respiratory rate was 16 breaths/min, and the oxygen saturation was 99%. The blood pressure in both upper arms was 135/80 mmHg. The laboratory examinations indicated no infection. Furthermore, he did not have a specific genetic or family history, nor any relevant employment, social, or exposure history.

### Diagnostic evaluation

**Fig. 1:**
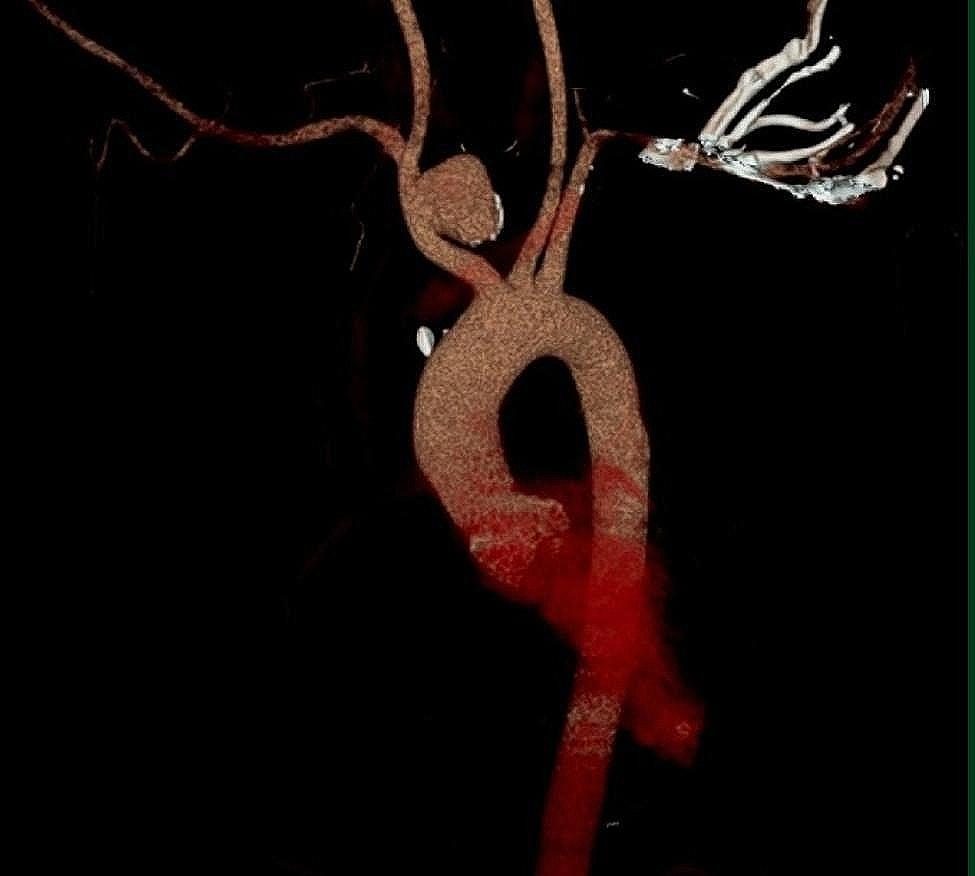
Preoperative CTA imaging and three-dimensional reconstruction revealed a 3.6*2.4 cm saccular aneurysm of the innominate artery that involved the right proximal subclavian and common carotid arteries

The physical examination revealed that the patient was 172 cm in height with a 73 kg weight. The pulse in the right radial artery was palpable but weaker than that of the left. The systolic brachial pressure was 110 and 125 mmHg in the right and left arms, respectively. Chest radiography showed an abnormal shadow on the upper mediastinum, while computed tomographic angiography (CTA) and three-dimensional reconstruction revealed a 3.6*2.4 cm saccular aneurysm of the innominate artery involving both the proximal right subclavian and common carotid arteries. No mural thrombus and neurological problems were observed and the patient did not complain of arm claudication. Gadolinium-enhanced magnetic resonance angiography indicated that the circle of Willis was intact and there was no notable intracranial carotid artery stenosis. Moreover, the coronary angiogram and echocardiogram indicated no abnormalities (Fig. 1).

### Therapeutic intervention

**Fig. 2:**
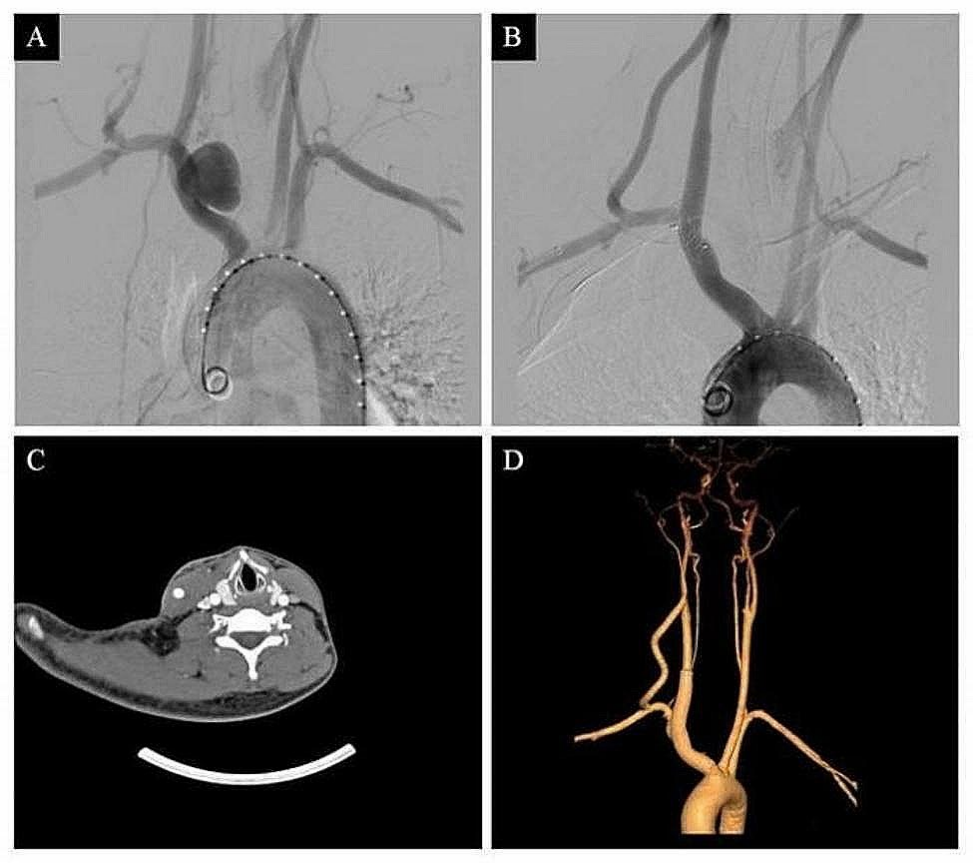
Representative images of (**A**) Intraoperative aortic arch angiography confirming the presence of a saccular aneurysm of the innominate artery involving the right proximal subclavian and common carotid arteries. (**B**) A combined endovascular and open-repair technique was used to cover the aneurysm, thereby avoiding the bleeding or endoleak. (**C**) Postoperative transverse computed tomography slide showing patency of both the right vertebral and graft arteries. (**D**) Postoperative follow-up CTA three-dimensional reconstruction of the aortic arch showing patency of the stent and right vertebral artery without endoleak or displacement

A right supraclavicular incision was made under general anesthesia and the right common carotid artery (RCCA) and right subclavian artery (RSA) were dissected. The right external carotid artery was carefully debranched, with transposition onto the RSA and an 8-mm woven Dacron graft insertion. A percutaneous 8Fr sheath was inserted in the right common femoral artery and a percutaneous 5Fr sheath was placed in the right brachial artery *via* a surgical cut down with distal clamping. After systemic heparinization (0.5 mg/kg), a pigtail was positioned in the aortic arch *via* the femoral access, and a pseudoaneurysm in the IA associated with the bifurcation was visible on subtraction angiography (Fig. 2A). No other aneurysms or notable stenoses were found. The Dacron graft through RSA to the external carotid artery was confirmed after contrast injection. Then, a 25 cm-long 12Fr sheath proximal to the IA and a 13*100-mm covered stent graft close to the aneurysm entrance by accessing percutaneously through the right groin. An occluder was inserted into the right brachial sheath to block the RSA at its origin. Subtraction angiography demonstrated the patency of the stent and total exclusion of the IAA, while antegrade opacification showed that the flow in the right vertebral artery was good (Fig. 2B).

### Follow-up and outcomes

The operation went smoothly without any complications, and postoperative transverse computed tomography showed prosthetic graft patency with no significant displacement of the stent. Furthermore, there were no vessel anastomosis stenosis and pseudoaneurysm, and both the right vertebral and graft arteries indicated patency. (Fig. 2C). The patient was discharged seven days after surgery and was prescribed 100 mg of Bay aspirin enteric-coated tablets and 10 mg of Rivaroxaban daily. The 1-year follow-up CTA showed no alterations in the aneurysm and no sign of vessel anastomosis stenosis and pseudoaneurysm in the prosthetic graft (Fig. 2D). Moreover, there were no other signs of endoleak, neurological complications, or limb ischemia.

## Discussion

Innominate artery aneurysms (IAA) are very rare, account for only 1% of peripheral aneurysms, and are predominantly caused by atherosclerosis [5, 6]. Most patients are asymptomatic and if not diagnosed at an early stage, the aneurysm gradually enlarges to compress the surrounding nerves, blood vessels, and trachea, and in severe cases, may cause serious life-threatening complications, such as rupture embolism [7]. Therefore, to avoid these serious consequences, aneurysms should be treated early. Traditional open aneurysm repair involves resection *via* median sternotomy or posterolateral thoracotomy. Then, the subclavian artery is reconstructed by the implantation of a prosthesis from the ascending aorta. Currently, open aneurysm repair remains the preferred treatment for IAA [2]. However, an 11% incidence of perioperative death following IAA repair has been reported and 18% of the patients require prolonged ventilation [8]. Studies have shown that vascular surgery combined with preoperative angiography and meticulous surgical planning by dedicated teams can improve surgical success. Furthermore, in stable patients, endovascular and hybrid procedures should be preferred [9].

The patient was hospitalized with chest discomfort and subsequent examination revealed a 3.6*2.4 cm aneurysm at the bifurcation of the innominate artery. For this patient, traditional open surgery was difficult as there was a high probability of postoperative complications. The endovascular treatment of peripheral aneurysms requires < 10 mm of aneurysms neck on both sides for stable anchorage and the complete block of aneurysm blood flow, therefore, this mono-treatment was also challenging. As the aneurysm was situated in the bifurcation of the innominate artery with an inadequate anchoring zone, therefore, a hybrid operation was suggested to be most beneficial for this patient. This necessitated the deployment of the stent graft from the right CCA to the innominate artery, over the subclavian artery. A major concern with this procedure was the possibility of acute ischemia in the upper right arm and vertebral artery following stent grafting. Therefore, it was decided to complete the external carotid artery to subclavian artery diversion in the general operating room before moving the patient to the digital subtraction angiography room. Before deployment of the stent graft, CTA was performed to confirm that the basilar circulation was intact and the RSA was visible following contrast injection from the left vertebral artery. This also confirmed the use of the bypass surgery for preventing right arm ischemia. The covered stent graft (Viabahn 13*100 mm) was positioned to release the proximal part of the innominate artery, resulting in the location of the stent graft in the RCCA, thus ensuring blood flow to the right subclavian and vertebral arteries. In addition, to prevent the type I endoleak resulting from blood flow on the covered stent graft at the innominate artery bifurcation, an occluder (Abbott 14 mm) was used to block the subclavian artery at its origin.

The vertebral artery is an important part of the cerebral blood supply, providing blood to the brain through the vertebrobasilar artery system, which supplies about 40% of the brain’s posterior circulation. Vertebral artery dominance (VAD) is defined as the dominance of one vertebral artery in terms of diameter, blood flow rate, and blood supply capacity. VAD is mainly caused by stenosis or hypoplasia of the contralateral vertebral artery, resulting in the dominant artery supplying more blood to the brain. There is no consensus on which vertebral artery is dominant in the overall population. Based on the above data, it was inferred that preserving the right vertebral artery in this patient is more appropriate for the surgery, without evaluation of the superior vertebral artery to minimize the risk of postoperative stroke.

## Conclusion

In summary, in this case study, a hybrid surgical approach was used to treat the patient, which involved an artificial vessel bypass of the subclavian artery followed by placement of a covered stent, and occlude. This strategy can replace the traditional open surgery, thereby greatly reducing the surgical risk, and ensuring blood flow of the patient’s right subclavian and vertebral arteries. Furthermore, this technique also reduces the risk of postoperative ischemia of the right upper limb and stroke. Moreover, it has significant benefits for the patient, especially for those with general systemic conditions and aneurysms located at arterial bifurcations. However, the sample size of this case report is small, and a large multicenter study with a longer follow-up period is needed to further demonstrate the safety and efficacy of this procedure.

## Data Availability

No datasets were generated or analysed during the current study.
